# A New Incremental Learning Method Based on Rainbow Memory for Fault Diagnosis of AUV

**DOI:** 10.3390/s25154539

**Published:** 2025-07-22

**Authors:** Ying Li, Yuxing Ye, Zhiwei Zhang, Long Wen

**Affiliations:** 1 College of Power Engineering, Naval University of Engineering, No.177 Jiefang Road, Wuhan 430033, China; 2 School of Mechanical Engineering and Electronic Information, China University of Geosciences, No. 388 Lumo Road, Wuhan 430074, China; 3 Shenzhen Research Institute, China University of Geosciences, Shenzhen 518057, China

**Keywords:** intelligent fault diagnosis, incremental learning, rainbow memory, deep learning

## Abstract

Autonomous Underwater Vehicles (AUVs) are gradually becoming some of the most important equipment in deep-sea exploration. However, in the dynamic nature of the deep-sea environment, any unplanned fault of AUVs would cause serious accidents. Traditional fault diagnosis models are trained in static and fixed datasets, making them difficult to adopt in new and unknown deep-sea environments. To address these issues, this study explores incremental learning to enable AUVs to continuously adapt to new fault scenarios while preserving previously learned diagnostic knowledge, named the RM-MFKAN model. First, the approach begins by employing the Rainbow Memory (RM) framework to analyze data characteristics and temporal sequences, thereby delineating boundaries between old and new tasks. Second, the model evaluates data importance to select and store key samples encapsulating critical information from prior tasks. Third, the RM is combined with the enhanced KAN network, and the stored samples are then combined with new task training data and fed into a multi-branch feature fusion neural network. The proposed RM-MFKAN model was conducted on the “Haizhe” dataset, and the experimental results have demonstrated that the proposed model achieves superior performance in fault diagnosis for AUVs.

## 1. Introduction

Concerning strategies in marine resource exploration and environmental surveillance, Autonomous Underwater Vehicles (AUVs) have risen as an indispensable tool for oceanic discovery and development [1]. Despite their great value, these vehicles usually operate in highly complex marine environments where extreme factors like hydrostatic pressure, thermal gradients, and salinity variations can induce performance degradation or catastrophic failures. Such operational disruptions not only compromise mission reliability and efficiency but also entail significant economic liabilities and safety risks [2]. Consequently, the development of intelligent fault diagnosis systems for AUVs has emerged as a challenge in marine engineering, with both academic and long-term engineering significance [3].

In recent years, deep learning has demonstrated considerable success in fault diagnosis for complex electromechanical systems [4]. While conventional fault diagnosis paradigms predominantly rely on static models trained with fixed datasets, their efficacy diminishes in dynamically evolving operational envelopes [5]. As AUVs undertake increasingly complex missions in time-varying marine environments, new fault modes continuously emerge. When confronted with new failure signatures, traditional diagnostic frameworks necessitate labor-intensive data re-acquisition, model retraining, and hyperparameter recalibration [6]. These iterative procedures incur prohibitive computational costs and prolonged update cycles, rendering them unsuitable for real-time fault diagnosis in deep-sea exploration scenarios.

In this context, incremental learning has emerged as a paradigm-shifting methodology to address these challenges [7]. This adaptive learning mechanism enables continuous knowledge acquisition from new data while retaining previously acquired intelligence. Through incrementally updating model parameters, it achieves real-time state estimation and self-optimization, effectively countering environmental dynamics and performance drift in deep-sea deployments. Furthermore, its localized update architecture minimizes computational overhead, rendering it particularly advantageous for resource-constrained AUV platforms. Previous studies on incremental learning have shown encouraging progress. Schaul et al. [8] proposed an improved experience replay approach that better accounts for the importance of different training samples. They proposed an experience prioritization framework that more frequently replays critical transitions, thereby enhancing learning efficiency. Cohen et al. [9] proposed a matrix approximation-based sampling strategy preserving data sparsity patterns, which excels in uniform data distributions but struggles with sparse reward environments. In contrast, Rainbow Memory addresses these limitations by efficiently selecting the most representative replay samples that can significantly improve the sample utilization efficiency and can offer a superior solution for incremental learning’s sample replay mechanism.

To address the challenges of high retraining costs and prolonged model update cycles caused by dynamic marine environmental changes in AUVs, this study proposes an RM-MFKAN model that combines Rainbow Memory (RM)-based incremental learning with a modified Kolmogorov–Arnold Network. The model leverages the advantages of incremental learning to achieve efficient and accurate fault diagnosis for AUVs, providing an innovative and practical solution for intelligent fault detection. The key contributions are as follows.

(1) A new application of Rainbow Memory-augmented incremental learning to AUV fault diagnosis, enabling continuous knowledge consolidation through strategically reintroduced historical samples while assimilating new operational data and mitigating catastrophic forgetting.
(2) A new two-dimensional convolutional feature fusion network is developed to extract both global fault characteristics and local correlation features from AUV multi-sensor data, enabling effective feature-level integration.
(3) The integration of the MFKAN model as the diagnostic backbone, which has been validated on the ‘Haizhe’ AUV benchmark dataset. The results demonstrate its superior multi-modal feature fusion capabilities compared to baseline architectures.

The remainder of this paper is organized as follows. Section 2 reviews related work. Section 3 presents the RM-MFKAN methodology. Section 4 describes the experimental setup and results. Section 5 concludes the paper.

## 2. Related Work

### 2.1. AUV Fault Diagnosis

Recent advances in deep learning have led to significant improvements in intelligent fault diagnosis methods for AUVs [6]. To address the complexity of state signals in deep-sea environments, Wu et al. [10] developed a multi-channel fully convolutional neural network-based fault diagnosis algorithm. By integrating multi-sensor signals to establish state-fault mapping relationships, their approach maintains a high accuracy of 91.4% even under missing data conditions. Xia et al. [11] innovatively combined bi-directional Gated Recurrent Units (GRUs) with spatial attention mechanisms for AUV fault diagnosis. Their method employs data attention for dynamic decorrelation and GRUs for temporal feature extraction, achieving exceptional detection performance on field test data from the “Qianlong-2” AUV of the South China Sea. Pei et al. [12] proposed a new NAS-based framework for AUV fault diagnosis that achieves breakthrough performance with a second-level architecture search on a single GPU. For handling the complexity and time-varying nature of large-scale multivariate time series (MTS) data, Xia et al. [13] developed a Hybrid Feature Adaptive Fusion Network (HFAF). This approach utilizes D-CNN and D-RNN to extract spatiotemporal information while employing attention mechanisms to eliminate redundancy across multi-scale features. In response to small-sample scenarios in AUV datasets, Gao et al. [14] integrated physical models with Generative Adversarial Network (GAN), incorporating physical constraints during GAN training to achieve 98.4% diagnostic accuracy with limited samples. Xu et al. [15] investigated an evidential reasoning method that can combine the multi-source fault feature information extracted from vibration signals to effectively reduce the uncertainty in diagnosis. Liu et al. [16] studied a multi-sensor cross-domain fault diagnosis method that can significantly improve the robustness of the fault detection to variations of operating environments. However, while these studies have achieved good results on the fault diagnosis of marine equipment, when the deep-sea environment changes, the data distribution of samples could change, leading to re-training in these situations.

### 2.2. Incremental Learning-Based Fault Diagnosis

Incremental learning has emerged as a prominent research focus in machine learning due to its demonstrated efficacy in mitigating catastrophic forgetting [17]. Recent studies have developed innovative solutions to address this challenge in deep learning-based multi-task fault diagnosis scenarios. Yin et al. [18] pioneered an approach combining Elastic Weight Consolidation (EWC) with Deep Residual Shrinkage Networks (DRSNs), establishing an effective framework for pressure relief valve fault diagnosis. Xia et al. [19] advanced the field through their Multi-Scale Knowledge Distillation with Label Smoothing system for incremental rotating machinery fault diagnosis, which employs knowledge distillation for model knowledge preservation and label smoothing to reduce model overconfidence, demonstrating robust performance in complex mechanical systems with three critical components.

Wang et al. [20] employed knowledge distillation to retain learned knowledge while employing shared classifiers across various operational scenarios to create a unified diagnostic architecture, achieving exceptional performance in rolling bearing fault diagnosis. Xu et al. [21] proposed the Causality-guided Broad Learning Model, which incorporates global and multi-scale local causal features. Model updates via weight expansion/modification enable effective incremental learning across two bearing datasets. Chen et al. [22] developed a Lifelong Learning Diagnosis Method that features a dual-branch aggregated residual network combined with sample retention. This method successfully overcomes catastrophic forgetting in bearing fault diagnosis with incremental fault types. Fu et al. [23] proposed a Dynamic Branch Layer-based Incremental Learning method. This method preserves historical knowledge using dedicated branch layers for each task and addresses model growth via innovative fusion structures. Although these studies can perform well in the incremental learning field by using knowledge distillation and dynamic architectural design methods, they may not be proficient in systematic preservation and the utilization of historically significant diagnostic information.

Replay-based incremental learning methods have demonstrated remarkable success across computer vision and natural language processing domains by strategically storing and replaying critical samples or features from historical data, thereby effectively mitigating catastrophic forgetting during new task acquisition. This paradigm has recently gained substantial traction in fault diagnosis applications, with several notable methodological advances. Hayes et al. [24] developed the REMIND framework, which introduced two key innovations: feature representation matching and an advanced replay mechanism. Their approach employs product quantization to compress and store feature representations from previous tasks, subsequently replaying these compressed features during new task learning to reinforce prior knowledge. Douillard et al. [25] proposed PODNet, incorporating spatial distillation loss and multi-agent vector representations to maintain an optimal balance between new task learning and old-task retention. This architecture demonstrates particular efficacy in long-term, small-scale incremental learning scenarios, significantly reducing forgetting rates. In mechanical fault diagnosis, Chen et al. [26] introduced an Incremental Fault Diagnosis with Bias Correction (IFD-BiC) method. Their solution combines distillation loss with cross-entropy optimization while employing both sample replay and a dedicated bias correction layer to address class imbalance between new and old categories, achieving superior diagnostic accuracy in diesel engine applications. For bearing fault diagnosis, Fu et al. [27] implemented a Maximum Dissimilarity Sampling (MDS) strategy that iteratively selects the most representative samples in feature space, ensuring comprehensive coverage of class distributions. Shen et al. [28] addressed catastrophic forgetting in imbalanced rotating machinery datasets through dynamic branch layer fusion, where dedicated branch layers preserve old task knowledge while fusion structures accommodate model expansion. Yan et al. [29] advanced wavelet transform-based system-level fault diagnosis through Forward and Backward Compatible representation Incremental Learning (FSCIL). Their method distills representative diagnostic knowledge into a reverse memory bank, providing robust protection against catastrophic forgetting. Based on the merit of replay-based incremental learning methods, this work develops a new RM-MFKAN model that can continuously adapt to new scenarios while preserving previously learned diagnostic knowledge.

## 3. Methodology

**Figure 1:**
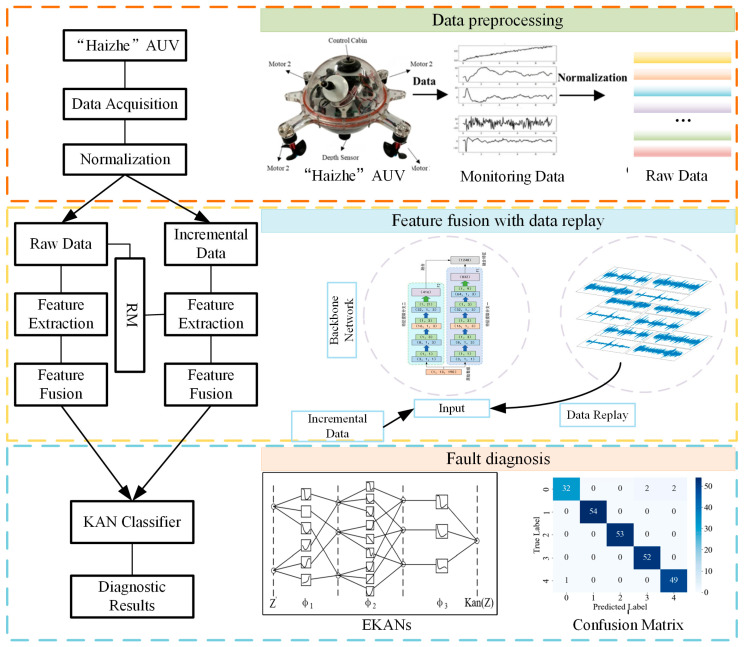
The structure of AUV fault diagnosis using RM-MFKAN model.

This research presents the RM-MFKAN model, an innovative framework that synergistically combines Rainbow Memory (RM)-based incremental learning with an enhanced Kolmogorov–Arnold Network (KAN) architecture. As illustrated in Figure 1, the model employs Rainbow Memory to analyze data characteristics and temporal patterns, which can establish the precise boundaries between old and new tasks through advanced feature-space partitioning. Following task reintroduction, the proposed RM-MFKAN implements a hierarchical selection process that evaluates samples based on informational significance, feature-space representativeness, and temporal relevance to construct an optimal memory bank of critical historical examples. Then, these preserved samples are integrated with new task data through a multi-branch convolutional network featuring parallel spatial–temporal feature extractors and cross-branch attention mechanisms, enabling comprehensive feature fusion across multiple abstraction levels. Third, the enhanced KAN component subsequently performs fault diagnosis via adaptive basis function optimization and multi-scale correlation analysis. By maintaining historical feature representations during new task assimilation through this sophisticated architecture, the model can effectively eliminate catastrophic forgetting while demonstrating enhanced diagnostic robustness in dynamic operational environments, particularly when handling sequential fault patterns in complex systems. The framework’s ability to balance knowledge retention with adaptive learning represents a significant advance in incremental fault diagnosis methodologies.

### 3.1. Rainbow Memory Module

To ensure that stored memory samples exhibit both intra-class representativeness and inter-class discriminability, we propose a feature-space sampling strategy [30]. Samples near classification boundaries typically possess high discriminative power, while those near class distribution centers offer greater representativeness. Our method reconciles these dual requirements through strategic feature-space sampling. A key challenge lies in maintaining sample diversity.

To overcome this issue, we developed an effective computational alternative scheme, using the uncertainty of the classification model as a proxy for the estimation of relative positions in the discriminant feature space. The core premise builds upon the empirical observation that classification models demonstrate higher confidence for representative samples located near class centroids. Operationally, we quantify sample uncertainty through a perturbation-based analysis framework. For each sample, we generate augmented variants via stochastic transformations (color jittering, random cropping, and occlusion) and measure output variance across these perturbations. Following Gal et al. [31], we formalize this through Monte Carlo (MC) approximation of the posterior distribution under a defined perturbation prior. *x* denotes the original sample, $\tilde{x}$ denotes perturbed variants, *y* denotes the label, and *A* denotes the number of augmentations. The perturbation prior $p(y=\tilde{x}|x)$ is modeled as a uniform mixture over transformation parameters $f_{r}\left(⬚\right)$ in Equation (2), where $\theta_{r}$ represents hyperparameters for the *r*-th perturbation. The sample uncertainty with respect to perturbations is quantified from the approximate distribution as formalized in Equations (4) and (5). In these expressions, $u\left(x\right)$ represents the uncertainty measure for sample *x*, and $I_{c}$ is the indicator function. *T* is the sample number of the class *C*. Lower $u\left(x\right)$ values indicate samples residing in high-confidence regions of the model’s feature space.

(1) $$p\left(y=c|x\right)=\int_{\tilde{D}}p\left(y=c|{\tilde{x}}_{t}\right)p\left({\tilde{x}}_{t}|x\right)d{\tilde{x}}_{t}\approx \frac{1}{A}\sum_{t=1}^{A}p\left(y=c|{\tilde{x}}_{t}\right)$$

(2) $$\tilde{x}=f_{r}\left(x|\theta_{r}\right),r=1,...,R$$

(3) $$\tilde{x}∼\sum_{r=1}^{R}\omega_{r}*f_{r}\left(x|\theta_{r}\right)$$

(4) $$S_{c}=\sum_{t=1}^{T}I_{c}argmax(p(y=\hat{c}|\tilde{x_{t}}))$$

(5) $$u\left(x\right)=1-\frac{1}{T}\underset{c}{max}S_{c}$$

Building upon the study by Bang et al. [32], we implement a balanced memory allocation scheme that distributes a fixed number of memory slots (*k_c_* slots per class) uniformly across all observed classes (N “seen” classes). The algorithm proceeds through three key operations: First, after initial slot assignment, we compute uncertainty metrics for both streaming samples ($D_{t}^{S}$) and stored samples ($D_{t-1}^{M}$) in task t memory buffer. Second, we rank all candidate samples $D_{c}$ by their uncertainty scores. Finally, we select samples from the ordered list using systematic interval sampling (stride $|D_{c}|/k_{c}$), thereby ensuring optimal diversity in the memory bank. This sampling strategy produces several important properties. First, the resulting memory population spans a continuous spectrum from perturbation-robust to perturbation-sensitive samples. Second, it introduces perturbation-induced diversity to episodic memory. Third, it enhances both the representativeness and robustness of the memory bank for incremental learning. Crucially, the interval selection mechanism maintains coverage of the complete uncertainty distribution while preventing cluster formation in the feature space.

### 3.2. MFKAN Foundation Model

**Figure 2:**
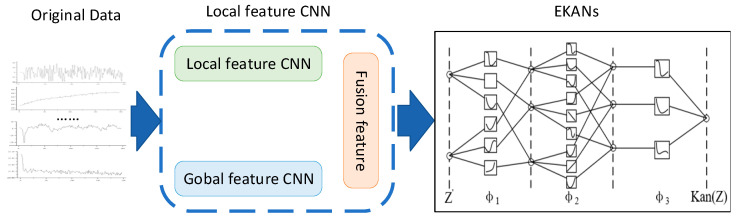
Network architecture of the MFKAN model.

The MFKAN architecture integrates a feature fusion network with an Enhanced Kolmogorov–Arnold Network (EKAN), as illustrated in Figure 2. The framework initiates with preprocessing operational state data collected from multiple AUV-mounted sensors, followed by systematic processing through a dual-branch feature fusion network. The global analysis branch extracts system-wide fault characteristics while the parallel local branch identifies component-specific signatures. The EKAN module then leverages its adaptive basis functions and dynamic topology optimization to perform a sophisticated analysis of the fused features, enabling precise fault pattern recognition through the hierarchical decomposition of complex feature interactions and nonlinear mapping of failure modes. This integrated architecture achieves robust diagnostic performance by simultaneously capturing both macroscopic system behavior and microscopic component anomalies while maintaining computational efficiency through EKAN’s advanced processing capabilities.

**Figure 3:**
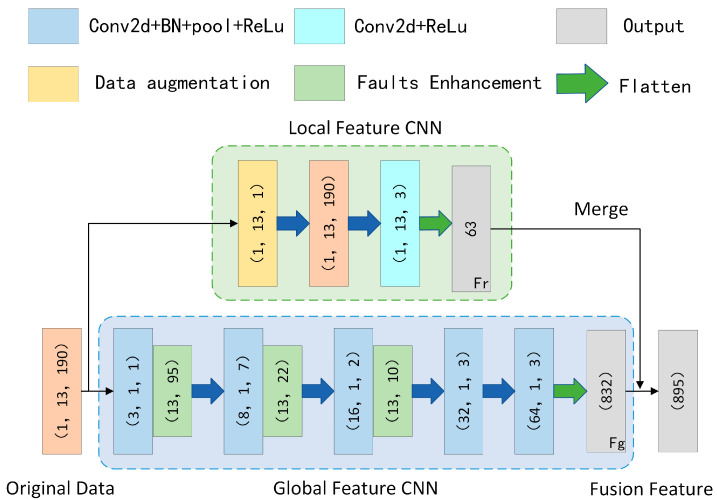
Architecture of the multi-sensor feature fusion network.

To address the issue of current AUV fault diagnosis methods in comprehensively extracting complete fault features from individual sensors, we propose a new multi-sensor feature fusion network. As illustrated in Figure 3, this architecture incorporates two specialized feature extraction modules: a global feature CNN designed to capture system-wide fault patterns across sensor arrays, and a local feature CNN that focuses on component-specific signatures from individual sensor streams. The integrated framework enables the synergistic analysis of heterogeneous sensor data through coordinated processing of both macroscopic system behavior and microscopic component anomalies, thereby overcoming the inherent constraints of single-sensor diagnostic approaches. This dual-pathway design ensures robust feature representation by simultaneously considering both the holistic system state and detailed local indicators while maintaining computational efficiency through parallel processing architecture. The global branch aggregates cross-sensor correlations to identify distributed fault patterns, whereas the local branch preserves high-resolution temporal and spatial features critical for precise fault localization, with subsequent feature fusion creating a comprehensive diagnostic representation that exceeds the capabilities of conventional single-sensor methods.

To effectively extract both global fault characteristics and local correlation features from the raw monitoring data of AUVs while achieving comprehensive feature fusion, we designed a new two-dimensional convolutional neural network (CNN) architecture employing one-dimensional kernels for global feature extraction. The global feature CNN utilizes a specialized 2D convolutional network with 1D kernels, incorporating carefully selected stride parameters to maintain both the independence and non-mixing of individual sensor data streams. The raw sensor data is structured as a two-dimensional array of dimensions H×W, where H represents the number of sensor types and W denotes the temporal length of sensor readings. The network architecture implements a sequential convolutional stack with kernel sizes of 1, 7, 2, 3, and 3, respectively, employing a consistent stride of 2, while the corresponding channel depths progress through 3, 8, 16, 32, and 64 layers. This optimized configuration enables the efficient extraction of system-wide fault features from AUV operational data, with the complete feature transformation process formally expressed in Equation (6).

(6) Fg=ReLu(GAP(BN(Conv2d1×n(Ci))))

The global feature extraction process transforms raw input data $C_{i}$ into comprehensive fault representations $F_{g}$, as formalized in Equation (6). To complement this global analysis, we developed a specialized local correlation network comprising two integrated components: a data augmentation module that emphasizes sensor-specific anomalies, and a local feature extractor that captures inter-sensor dependencies. The augmentation module employs global average pooling to identify anomalous sensor behaviors, subsequently amplified through sigmoid activation to highlight abnormal operational states. This targeted enhancement enables a more discriminative representation of localized fault patterns in AUV systems. The augmentation transformation is mathematically defined in Equation (7), where the adaptive weighting mechanism selectively intensifies diagnostically relevant sensor channels while suppressing normal operational signatures.

(7) $$C_{n}=C_{i}⊗\sigma \left(GAP\left(C_{i}\right)\right)$$

The data augmentation process yields enhanced sensor readings, where $C_{n}$ denotes the augmented data for the *n*-th sensor and $C_{i}$ represents the original measurements from the i-th sensor, with σ indicating the sigmoid activation function. Building upon this augmented representation, we developed a dedicated local feature extraction module to capture inter-sensor correlation patterns. This module analyzes localized dependencies across sensor arrays through a hierarchical convolution operation, systematically modeling both temporal and cross-channel relationships. The mathematical formulation for extracting these local correlation features is presented in Equation (8), which establishes a differentiable mapping from raw sensor measurements to discriminative fault signatures while preserving the physical interpretability of inter-sensor interactions.

(8) Fr=ReLu(Conv2dn×3(Ci))

The convolutional operation systematically captures inter-sensor correlations in AUV systems through kernel sliding across raw data streams. To holistically represent fault conditions, we performed a feature-level fusion of global fault characteristics and local correlation features, integrating their complementary diagnostic information. This fusion process, formally specified in Equation (9), generates comprehensive fault embeddings that preserve both system-wide patterns and localized inter-sensor dependencies.

(9) $$F_{o}=Flatten(F_{r})⊕Flatten(F_{g})$$

The feature fusion process combines local correlation features ($F_{r}$) with global fault characteristics ($F_{g}$) to generate comprehensive diagnostic representations. By integrating sensor-level anomalies with system-wide fault patterns at the feature level, the multi-sensor fusion network produces discriminative embeddings that enable both holistic state characterization and precise fault identification.

### 3.3. Loss Function

The cross-entropy loss function is a fundamental objective function in machine learning, particularly for multi-class classification tasks. It quantifies the dissimilarity between the predicted probability distribution and the true label distribution, serving as a critical optimization target during model training. In multi-class settings, the cross-entropy loss is derived from information theory, measuring the expected number of bits required to encode the true class labels using the predicted probabilities.

Formally, given a classification problem with *C* classes, let $y=(y_{1},y_{2},...,y_{C})$ denote the one-hot encoded ground-truth label, where *y_i_* = 1 if the sample belongs to class *i* and is 0 otherwise. Let $p=(p_{1},p_{2},...,p_{C})$ represent the predicted probability distribution, typically obtained via the softmax function applied to the model’s logits. The multi-class cross-entropy loss is then defined as in Equation (10).

(10) $$L\left(y,p\right)=-\sum_{i=1}^{C}y_{i}\log\left(p_{i}\right)$$

Due to the one-hot nature of *y*, this expression simplifies the negative log-likelihood of the true class, emphasizing the model’s confidence in the correct prediction. Minimizing this loss encourages the predicted probabilities to align closely with the true distribution, thereby improving classification accuracy.

The cross-entropy loss is widely favored in deep learning due to its well-behaved gradients, which facilitate efficient optimization, and its natural interpretation as a likelihood-based objective. By penalizing incorrect predictions more severely when the model is highly confident, it drives the learning process toward robust and discriminative feature representations. Consequently, it remains the standard choice for training neural networks in multi-class classification scenarios.

## 4. Experimental Study

### 4.1. AUV Dataset

The fault data was collected from navigation experiments conducted using Zhejiang University’s “Haizhe” AUV. The Haizhe AUV is a compact unmanned underwater platform equipped with a propulsion motor, depth sensor, and control module, as shown in Table 1. For monitoring purposes, the vehicle was instrumented with both depth sensors and inertial measurement units (IMUs). Table 2 details the sensor data specifications. Each experimental trial introduced a single fault condition lasting 1020 s. The Haizhe platform exhibits five operational states, named normal operation, depth sensor failure, increased payload, minor propeller damage, and severe propeller damage.

The data preprocessing pipeline begins with sample extraction from raw sensor streams using a sliding window approach with a fixed size of 1024 data points. This windowing operation facilitates local feature capture while generating representative segments for subsequent analysis. From the “Haizhe” dataset, we selected 13 sensor modalities (excluding temporal markers) that collectively encode the AUV’s operational states across diverse environmental conditions. Each sensor channel undergoes standardized processing: (1) uniform length adjustment to 190 data points through truncation of excess values or replication of terminal values for shorter sequences, ensuring dimensional consistency while preserving data continuity; (2) min–max normalization to the [−1, 1] interval to enhance comparability across heterogeneous sensors. The processed dataset is then randomly partitioned into training and testing subsets, maintaining a balanced representation of all fault conditions.

### 4.2. Experimental Setup

All experiments were conducted under identical environmental conditions to ensure comparability. The proposed RM-MFKAN model was implemented on a Windows 10 platform using Python 3.9 and PyTorch 2.2.1, with hardware acceleration provided by an NVIDIA RTX 2080Ti GPU and Intel^®^ Core™ i9-10900X CPU.

To maintain experimental rigor, we standardized hyperparameters across all evaluations using the “Haizhe” dataset, as detailed in Table 3. Specifically, the configuration employed included learning rate, batch size, and epoch number, as described below.

### 4.3. Incremental Learning Performance

“Inc 1” means that all the methods are trained using label 0 and 1. “Inc 2” means that all the methods conducted the incremental learning. “Inc 2” is based on “Inc 1”, and it adds the label 2 and 3. “Inc 3” is an incremental learning as well. It is based on “Inc 2” and adds the label 4.

To rigorously evaluate the performance of RM-MFKAN, we conducted comparative experiments against ten established incremental learning methods, namely Finetuning [33], EEIL [34], EWC [35], Freezing [36], LUCIR [37], LwF [38], LwM [39], Path Integral [40], RWalk [41], and iCaRL [7]. These experiments were performed using a publicly available AUV dataset under identical conditions to ensure fair comparison. As evidenced in Table 4, RM-MFKAN demonstrates superior performance in both classification accuracy and computational efficiency across all incremental phases. The model achieves perfect 100% accuracy in the initial phase, outperforming all baseline methods and validating its exceptional fault diagnosis capability. Remarkably, RM-MFKAN maintains 98.1% accuracy after the second incremental phase (a mere 1.9% decrease) and 96.6% after the third phase (only a 1.5% further decline), significantly surpassing the 97.3% peak accuracy of competing methods. These results collectively demonstrate RM-MFKAN’s robust ability to assimilate new fault categories while effectively mitigating catastrophic forgetting, highlighting its dual strengths in maintaining classification precision and adaptive learning capacity under continuous data integration scenarios. The consistent performance advantage across incremental phases underscores the model’s suitability for real-world deployment, where sustained diagnostic accuracy is paramount.

### 4.4. Ablation Study

Table 5 presents a systematic evaluation of different sampling strategies’ impacts on incremental learning performance. The study compares four methods, including simple random sampling, which selects samples uniformly from the population; reservoir sampling, a stream-processing algorithm that maintains fixed-size representative samples without prior knowledge of total data volume; ring-buffer sampling, where new data overwrites the oldest entries in a circular storage structure; and the proposed Rainbow Memory sampling method. Performance metrics reveal Rainbow Memory’s superior efficacy, achieving peak accuracy rates of 100%, 98.1%, and 96.6% across the three incremental phases, respectively. This represents a statistically significant improvement over conventional methods, demonstrating Rainbow Memory’s unique capability to preserve critical feature distributions while accommodating new data streams. The circular buffer technique showed particular limitations in later phases, with accuracy degradation suggesting insufficient protection against catastrophic forgetting. Reservoir sampling, while theoretically sound for streaming scenarios, proved less effective in maintaining long-term knowledge retention compared to the proposed method’s discriminative sampling approach. These findings substantiate Rainbow Memory’s dual advantages: maintaining representative exemplars from historical distributions while dynamically adapting to emerging data patterns—a crucial requirement for effective incremental learning in AUV fault diagnosis applications. The consistent performance across phases underscores the method’s robustness compared to conventional sampling baselines.

Table 6 illustrates the impact of varying random seeds on the incremental learning process. Classes were randomly partitioned and allocated across five tasks using distinct random seeds (1–3) to construct an incremental learning task configuration. The results reveal a non-monotonic relationship between the random seed value and model accuracy: as the seed increases from 1 to 3, incremental learning performance first rises and then declines. Consequently, a random seed of 2 was selected for the Blurry dataset to optimize accuracy.

Table 7 demonstrates the impact of varying episodic memory sizes (K) on the incremental learning process. As K increases from 100 to 500, the accuracy rates across all three incremental learning phases show consistent improvement. However, when K is further increased to 600, the performance in phases 2 and 3 begins to decline. Based on these observations, we selected K = 500 as the optimal memory size for our final experiments.

Table 8 presents a comparative analysis of how different data augmentation strategies affect incremental learning performance. The study evaluates three prominent techniques—CutMix (which synthesizes new samples by blending cropped regions from paired images), Cutout (which occludes random rectangular areas with uniform pixel values), and RandAugment (which provide details regarding where data supporting reported results can be found, including links to stochastic combinations of predefined transformations at varying intensities)—against a non-augmented baseline. Crucially, the baseline model consistently achieved higher accuracy rates (100%, 98.1%, and 96.6% across three incremental learning phases) than all augmented variants, suggesting that standard augmentation approaches may inadvertently compromise the preservation of learned features in incremental learning scenarios. This counterintuitive finding highlights the need for task-specific augmentation design in continual learning systems.

## 5. Conclusions

To address the practical application issues of high retraining costs and prolonged model update cycles caused by evolving fault types in AUVs, this paper proposes an RM-MFKAN-based fault diagnosis method. The approach first designs a multi-sensor feature fusion network to extract both global fault characteristics and local correlation features from AUVs by comprehensive feature-level integration. Second, a Rainbow Memory (RM) module is incorporated for incremental learning. The RM module calculates sample perturbations to select optimal samples for replay. Third, the fault diagnosis is implemented using an EKAN network. During incremental learning updates, the input samples include both new data and stored historical samples. This sample replay mechanism enables the model to acquire new knowledge while effectively retaining critical learned information. Several experiments on AUV datasets have demonstrated RM-MFKAN’s superior performance. The performance of RM-MFKAN achieves 98.1% classification accuracy across all fault categories after the first incremental learning and can maintain 96.6% accuracy after the second incremental update. These results validate RM-MFKAN’s exceptional capability to significantly mitigate catastrophic forgetting while maintaining the robust recognition of novel fault types. In the future, the proposed method can be conducted on a real-world AUV system, and the real-world experiments can be used to validate the proposed RM-MFKAN.

## Figures and Tables

**Table 1:** Sensor data of “Haizhe” AUV dataset.

Name	Description	Name	Description
Time	Absolute time (s)	Yaw	Yaw angle (degree)
PWM1-4	Control time (ms)	a_x	Acceleration of *X*-axis (m/s^2^)
Depth	Diving depth (ms)	a_y	Acceleration of *Y*-axis (m/s^2^)
Press	Pressure value (Pa)	a_z	Acceleration of *Z*-axis (m/s^2^)
Voltage	Supply voltage (V)	w_row	Row angular Acceleration (degree/s)
Roll	Roll angle (degree)	w_pitch	Pitch angular Acceleration (degree/s)
Pitch	Pitch angle (degree)	w_yaw	Yaw angular Acceleration (degree/s)

**Table 2:** Details of “Haizhe” dataset.

Fault State	Label	Dataset	Train	Test
Normal	0	182	146	36
Load increase	1	268	214	54
Depth Sensor fault	2	266	213	53
Slight damage to propeller	3	260	208	52
Severe damage to propeller	4	249	199	50

**Table 3:** Hyperparameter settings for model training.

Hyperparameters	lr	Optimizer	Loss Function	Batch	Epochs
Set	0.001	Adam	Cross-entropy	128	200

**Table 4:** Incremental learning performance of RM-MFKAN on AUV dataset.

	Inc 1		Inc 2		Inc 3	
	ACC (%)	Time (s)	ACC (%)	Time (s)	ACC (%)	Time (s)
Finetuning	97.8	2.67	95.7	2.49	93.0	2.26
eeil	97.8	2.58	88.3	2.43	64.1	2.28
Ewc	97.8	2.38	97.2	2.35	96.3	2.32
Freezing	97.8	2.59	97.3	2.48	96.0	2.43
Lucir	100	3.45	95.7	2.68	87.2	2.38
Lwf	97.8	2.68	86.9	2.59	80.4	2.52
path_integral	97.8	2.72	92.9	2.66	88.8	2.46
RWalk	97.8	2.69	96.8	2.63	94.6	2.58
Icarl	97.8	2.54	96.3	2.47	89.5	2.34
RM-MFKAN	100	2.32	98.1	2.24	96.6	2.16

**Table 5:** The impact of different sampling strategies on the incremental learning process.

Sampling Strategy	Inc 1 ACC (%)	Inc2 ACC (%)	Inc3 ACC (%)
Simple Random Sampling	100	97.0	95.7
Reservoir Sampling	100	96.7	95.0
Ring Buffer Sampling	100	97.1	95.5
Rainbow Memory	100	98.1	96.6

**Table 6:** The impact of different random seeds on the incremental learning process.

Random Seed for the Blurry Dataset	Inc 1 ACC (%)	Inc2 ACC (%)	Inc3 ACC (%)
Rnd_seed = 1	95.5	89.7	87.1
Rnd_seed = 2	100	98.1	96.6
Rnd_seed = 3	94.1	88.4	84.9

**Table 7:** The impact of K on incremental learning.

K	Inc 1 ACC (%)	Inc2 ACC (%)	Inc3 ACC (%)
100	77.1	62.8	59.2
200	97.1	93.4	90.6
300	98.1	95.3	92.6
400	99.0	96.5	93.4
500	100	98.1	96.6
600	100	97.3	95.5

**Table 8:** Impact of different data augmentation methods on incremental learning.

Data Augmentation Methods	Inc 1 ACC (%)	Inc2 ACC (%)	Inc3 ACC (%)
None	100	98.1	96.6
cotmix	100	97.4	96.2
cutout	100	97.9	96.6
Randaugment	100	97.1	96.2

## Data Availability

The data presented in this study are available on request from the corresponding author.
